# The association between visceral adiposity index and female stress urinary incontinence: the NHANES 2013 to 2018

**DOI:** 10.1097/MD.0000000000045610

**Published:** 2025-11-14

**Authors:** Dakai Sun, Caie Hou, Xingcheng Zhu, Botao Xie, Haoyang He, Yanqi Wang

**Affiliations:** a Department of Urology Surgery, The Second People’s Hospital of Qujing, Yunnan, China; b Department of the Party Committee, The First People’s Hospital of Qujing, Yunnan, China; c Department of Clinical Laboratory, The Second People’s Hospital of Qujing, Yunnan, China.

**Keywords:** female, NHANES, stress urinary incontinence, visceral adiposity index

## Abstract

Obesity is an independent risk factor for stress urinary incontinence (SUI) in women. The visceral adiposity index (VAI) has recently been used as a reliable index to assess visceral fat accumulation and dysfunction. The objective of this research was to examine the correlation between VAI and SUI among adult females. Our study is a cross-sectional study derived from the National Health and Nutrition Examination Survey. A total of 5938 females were enrolled, and all participants had complete VAI and SUI data. We analyzed weighted multivariable logistic regression to assess the independent association between VAI and SUI. In addition, we also used subgroup analyses and interaction tests to examine the stability of this result. Among 5938 female adult participants, 2438 of whom were SUI, there was a positive association between VAI and SUI. In the fully adjusted model, each 1 unit increase in VAI index was associated with a 3% increase in the risk of developing SUI [1.03 (1.01–1.06)]. Compared to those in the lowest quartile, participants in the highest quartile of VAI exhibited a 57% higher prevalence of SUI [1.57 (1.33–1.86)]. The observed positive correlation was particularly prominent among individuals aged 20 to 40, often vigorous recreational activities, nondiabetic, Mexican American, and non-Hispanic black populations. VAI and SUI have a positive relationship among females in the United States.

## 1. Introduction

There are various risk factors for stress urinary incontinence (SUI), like age, fertility, pelvic organ prolapse, obesity, family history, etc.^[1]^ According to statistics, about 23% to 45% of women worldwide suffer from different degrees of urinary incontinence, of which approximately 50% are SUI.^[2]^ SUI is a common disease among adult females, which can cause significant disturbance to the patient’s daily activities and socialization and even cause great psychological stress.^[3]^ With the increase in the number of older adults globally, the incidence of SUI gradually increases each year.^[4]^

Obesity is already a significant public health issue, and its prevalence and associated problems continue to increase annually.^[5]^ SUI is substantially more likely to occur in obese women, and weight loss is associated with improvement and remission of SUI.^[6]^ Previously, the only common obesity indexes related to the prevalence of SUI were BMI and WC. Still, they could not differentiate between subcutaneous fat and visceral fat, and the accumulation of visceral fat produces most of SUI.^[7]^ The pathophysiologic link between female SUI and obesity is usually directly attributed to the increased intra-abdominal pressure and sagittal diameter of the abdominal cavity resulting from weight gain.^[8]^ This hyperpressure is thought to damage the pelvic floor, leading to musculotendinous and neurological damage.^[9]^ At the same time, the increase in abdominal pressure is transmitted to the bladder, leading to bladder hyperpressure.^[10]^ The VAI index is derived based on different genders, BMI, WC, HDL-C, and TG calculations and is used to assess the degree of visceral adiposity; it was initially suggested by Amato et al.^[11]^ The BMI is a widely employed metric for assessing obesity. However, it is subject to various influencing factors and may not provide an accurate evaluation for specific individuals who are obese. On the other hand, it and waist circumference (WC) are utilized to evaluate abdominal obesity, but they fail to account for subcutaneous and visceral fat.^[12]^ In contrast, the modified VAI offers a more comprehensive assessment of visceral fat levels.^[13]^

Thus, the present study examined the correlation between VAI and SUI in a population of females aged 20 years and above. This investigation included analyzing data from the NHANES.

## 2. Methods

### 2.1. Study population and date

**Figure 1.**
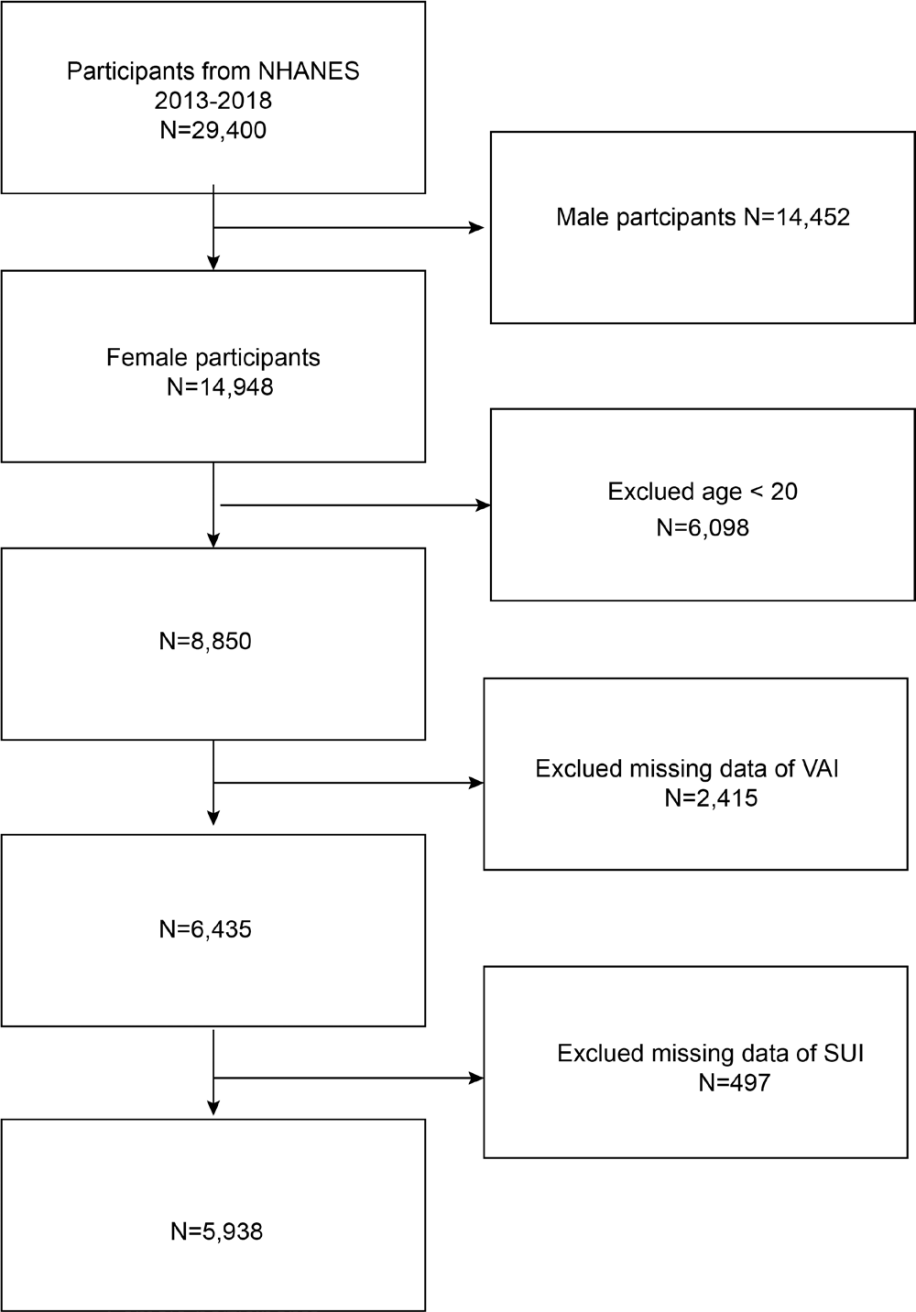
Flow chart of participants selection. A total of 29,400 participants were enrolled at first, and after the exclusion of male participants (n = 14,452), age <20 yr (n = 6098), incomplete data about VAI (n = 2415), and SUI (n = 497). SUI = stress urinary incontinence, VAI = visceral adiposity index.

All the data for our study came from the NHANES database.^[14]^ The research data included in our study consisted of 3 cycles of NHANES from 2013 to 2018. Also, our data contains complete VAI and SUI data for all participants. Our study initially included 29,400 participants, and we rule out male participants (n = 14,452), minor females (aged < 20 years) (n = 6098), Incomplete data for VAI (n = 2415), and SUI (n = 497). The final 5938 participants were enrolled in our study (Fig. 1).

### 2.2. Visceral adiposity index

The VAI assesses visceral obesity, a composite index based on WC, BMI, TG, and HDL-C.^[15]^ A more excellent value of the VAI index reflects a higher degree of visceral obesity. The formula for calculating female VAI for this study is WC/(36.58 + (1.89*BMI))*(TG/0.81)*(1.52/HDL-C).^[16]^ TG and HDL-C were millimoles per liter (mmol/L), whereas WC was computed in centimeters (cm). In our research, the variable VAI was treated as a continuous measure, and participants were grouped into quartiles based on their VAI level for further analysis.

### 2.3. Stress urinary incontinence

Information from the SUI questionnaire was administered to all participants. Data obtained from the Urology file (Questionnaire: KIQ042). Questions from investigators like “Leak urine during physical activities?’’. If the answer is “1” for yes, it means suffering from SUI.^[17,18]^

### 2.4. Covariates of interest

The covariates we included were age, race, the ratio of family income to poverty (PIR), education level, diabetes, smoked ≥100 cigarettes, marital status, high blood pressure, total cholesterol, and vigorous recreational activities.

### 2.5. Statistical analysis

Data were separated into quartiles and examined using a weighted Student *t* test and chi-square test to evaluate the variations in participant demographics. To explore the relationship between VAI and SUI, the research employed multivariable regression models, incorporating the NHANES complex sample methodology. No adjustments were made in Model 1, and Model 2 was adjusted for age and race. Finally, Model 3 was adjusted for age, race, PIR, education level, diabetes, smoked ≥100 cigarettes, marital status, high blood pressure, total cholesterol, and vigorous recreational activities. The relationship between VAI and SUI was analyzed using subgroup analyses for populations in different states, including age, race, high blood pressure, vigorous recreational activities, and diabetes. The study included interaction testing to evaluate whether the identified associations were constant across different subgroups. The analyses were performed using R (version 4.3.2) or Empowerstats. The statistical significance was established at a significance level of *P* <.05.

## 3. Results

### 3.1. Baseline characteristics

We included a total of 5938 female participants with a mean age of 48.05 ± 7.52 years, of whom 41.05% had SUI. The average VAI is 5.65 ± 0.83, and the VAI quartiles 1 to 4 were (0.21–1.05), (1.06–1.74), (1.75–3.04), and (3.05–28.29), respectively. Participants in the higher quartiles of VAI had higher rates of SUI (Q1: 33.00%; Q2: 38.09%; Q3: 42.51%; Q4: 50.74%; *P* <.001). In the VAI quartiles, differences with statistical significance were observed in age, race, PIR, education level, diabetes, smoked ≥100 cigarettes, WC, BMI, TG, HDL-C, high blood pressure, total cholesterol, and vigorous recreational activities (all *P* <.05). Participants with the highest VAI in Q4 tend to be older females, have less than high school education, have less vigorous recreational activities, and smoke more often. Participants with a higher VAI were more likely to have a higher likelihood of diabetes, high blood pressure, BMI, WC, total cholesterol, triglycerides, HDL-C, and SUI compared with the lowest VAI quartile. We also found that participants with higher VAI typically had higher BMI, WC, and TG (Table 1).

**Table 1:** Basic characteristics of participants by visceral adiposity index among U.S. female adults.

VAI	Q1 (0.21–1.05)	Q2 (1.06–1.74)	Q3 (1.75–3.04)	Q4 (3.05–28.29)	*P*-value
	N = 1482	N = 1486	N = 1484	N = 1486	
Age (year)	45.14 ± 17.49	48.43 ± 17.49	52.18 ± 17.45	52.71 ± 15.98	<.0001
Race, (%)					
Mexican American	(8.99)	(13.59)	(19.03)	(21.33)	<.0001
Other Hispanic	(8.59)	(11.84)	(12.48)	(13.32)	
Non-Hispanic White	(37.19)	(36.20)	(39.27)	(39.77)	
Non-Hispanic Black	(28.67)	(25.24)	(16.40)	(10.50)	
Other Race	(16.57)	(13.12)	(12.82)	(15.07)	
Education level, (%)					
Less than high school	(11.43)	(19.18)	(23.35)	(26.04)	<.0001
High school or GED	(17.17)	(20.39)	(23.48)	(23.28)	
Above high school	(71.40)	(60.43)	(53.17)	(50.67)	
Marital status, (%)					
Married or living with partner	(53.28)	(53.36)	(54.79)	(57.40)	.084
Living alone	(46.72)	(46.64)	(45.21)	633 (42.60)	
PIR	2.83 ± 1.68	2.61 ± 1.61	2.40 ± 1.58	2.32 ± 1.52	<.0001
Smoked ≥100 cigarettes, (%)					
Yes	(29.89)	(32.30)	(33.06)	(39.84)	<.0001
No	(70.11)	(67.70)	(66.94)	(60.16)	
Diabetes, (%)					
Yes	(6.63)	(11.04)	(17.75)	(26.72)	<.0001
No	(93.37)	(88.96)	(82.25)	(73.28)	
High blood pressure, (%)					
Yes	(24.14)	(35.13)	(42.65)	(47.85)	<.001
No	(75.86)	(64.87)	(57.35)	(52.15)	
Vigorous recreational activities, (%)					
Yes	(33.13)	(21.40)	(14.04)	(11.84)	<.001
No	(66.87)	(78.60)	(85.96)	(88.16)	
HDL-C (mmoL/L)	1.91 ± 0.44	1.60 ± 0.32	1.39 ± 0.27	1.13 ± 0.24	<.001
Total cholesterol (mmoL/L)	2.34 ± 0.09	2.33 ± 0.09	2.34 ± 0.10	2.35 ± 0.10	.0004
Triglycerides (mmoL/L)	0.70 ± 0.20	1.10 ± 0.25	1.58 ± 0.35	2.83 ± 1.08	<.001
BMI	26.40 ± 6.87	29.51 ± 7.52	31.50 ± 7.77	32.30 ± 7.14	<.001
Waist circumference, (cm)	88.95 ± 15.30	97.47 ± 16.42	102.68 ± 16.44	106.04 ± 15.54	<.001
SUI, (%)					
Yes	(33.00)	(38.09)	(42.51)	(50.74)	<.001
No	(67.00)	(61.91)	(57.49)	(49.26)	

Mean ± SD for continuous variables: the *P*-value was calculated by the weighted linear regression model; (%) for categorical variables: the *P*-value was calculated by the weighted chi-square test.

BMI = body mass index, GED = general educational developmented, PIR = the ratio of family income to poverty, Q = quartile, SD = standard deviation, SUI = stress urinary incontinence.

### 3.2. Association between VAI and SUI

In Table 2, the association between VAI and SUI is shown. We found an association between a higher VAI index and an increased likelihood of SUI morbidity. All models in our study found a positive correlation between VAI and SUI. In model 3, per 1 unit increase in the VAI, the risk of SUI increased by 3% (OR = 1.03, 95% CI: 1.01–1.06). In addition, the association between them is still statistically significant after we transformed the VAI into quartiles. Table 2 shows that the probability of SUI was 57% higher in Q4 compared to the VAI index quartile (Q1).

**Table 2:** Associations between visceral adiposity index and stress urinary incontinence.

VAI	Crude model (Model 1)	Minimally adjusted model (Model 2)	Fully adjusted model (Model 3)
VAI/OR (95% CI)			
Continuous	1.07 (1.05–1.09) <0.0001	1.04 (1.02–1.07) 0.0001	1.03 (1.01–1.06) 0.0117
Categories			
Quartile 1	1.0	1.0	1.0
Quartile 2	1.25 (1.07–1.45) 0.0042	1.16 (0.99–1.35) 0.0667	1.12 (0.95–1.32) 0.1707
Quartile 3	1.49 (1.28–1.73) <0.0001	1.24 (1.06–1.45) 0.0064	1.19 (1.01–1.41) 0.0395
Quartile 4	2.09 (1.80–2.42) <0.0001	1.71 (1.46–2.00) <0.0001	1.57 (1.33–1.86) <0.0001

Model 1: no covariates were adjusted. Model 2: age and race were adjusted. Model 3: age, race, education level, marital status, the ratio of family income to poverty, smoked ≥100 cigarettes, diabetes, high blood pressure, vigorous recreational activities were adjusted.

BMI = body mass index, CI = confidence interval, OR = odds ratio, Q = quartile, SUI = stress urinary incontinence, VAI = visceral adiposity index.

### 3.3. Subgroup analyses

As indicated in Table 3, we conducted subgroup analyses to assess whether there may be a connection between VAI and SUI in various groups. The connection between the SUI and ages, races, vigorous recreational activities, and diabetes display significant interactions (*P* for interaction <.05). Meanwhile, a positive relationship in age (20–40), Mexican American, non-Hispanic black, vigorous recreational activities, and nondiabetic participants.

**Table 3:** Subgroup analysis of the association between VAI and SUI.

SUI	OR (95% CI)	*P* for interaction
Age (year)		
20–40	1.10 (1.05–1.15)	.0009
40–60	0.99 (0.97–1.02)	
60–80	1.04 (1.00–1.08)	
Race, (%)		
Mexican American	1.09 (1.02–1.16)	.0087
Other Hispanic	0.99 (0.96–1.02)	
Non-Hispanic White	1.01 (0.97–1.05)	
Non-Hispanic Black	1.12 (1.03–1.21)	
Other Race	1.04 (0.98–1.10)	
High blood pressure		
Yes	1.03 (1.00–1.06)	.5316
No	1.02 (0.98–1.05)	
Vigorous recreational activities		
Yes	1.12 (1.04–1.20)	.0113
No	1.02 (0.99–1.04)	
Diabetes, (%)		
Yes	1.00 (0.97–1.02)	.0034
No	1.06 (1.03–1.09)	
Smoked ≥100 cigarettes, (%)		
Yes	1.03 (1.00–1.07)	.2152
No	1.02 (0.99–1.05)	
Education level, (%)		
Less than high school	1.01 (0.98–1.04)	.2286
High school or GED	1.00 (0.97–1.02)	
Above high school	0.99 (0.96–1.02)	
Marital status, (%)		
Married or living with partner	1.01 (0.98–1.04)	.1357
Living alone	1.02 (0.98–1.06)	

Age, race, high blood pressure, vigorous recreational activities, PIR, smoked ≥100 cigarettes, marital status and education level were adjusted.

BMI = body mass index, CI = confidence interval, OR = odds ratio, PIR = the ratio of family income to poverty, Q = quartile, SUI = stress urinary incontinence, VAI = visceral adiposity index.

## 4. Discussion

5938 participants in our cross-sectional study showed that as the VAI index increased, the likelihood of SUI increased, especially in younger (20–40 years), often vigorous recreational activities, and nondiabetic Mexican American and non-Hispanic black populations. The present results indicated that controlling the growth of the VAI index may help reduce the incidence of SUI.

The International Diabetes Federation recommends using MRI or CT scans to assess visceral fat accumulation, but these tests are expensive and complex.^[19]^ Amato et al proposed the VAI, for assessing visceral obesity leading to associated diseases.^[11,20]^ VAI is a good predictor of visceral fat accumulation, leading to cardiovascular disease, hypertension, insulin resistance and thyroid function. Peng et al found that VAI was significantly associated with the incidence of CKD in the elderly population and, in particular, had an excellent predictive value for early CKD.^[21]^ Hou et al reported a novel predictor for kidney stone prevention by establishing a positive correlation between the VAI index and kidney stone prevalence.^[22]^ VAI levels were significantly associated with hypertension and prehypertension in the general Chinese population.^[23]^ Through our study, we agree that VAI is a pretty predictive indicator for evaluating visceral disease.

The mechanism by which visceral fat causes SUI in women is not well understood in research, and the following reasons may exist: visceral fat accumulation increases pelvic pressure, and bladder pressure is more significant than urethral closure pressure, leading to SUI.^[24]^ Resting intravesical bladder pressure is higher in abdominal obesity in women and generates more pressure during maximal coughing.^[25]^ The pro-inflammatory activity of adipokines produced by the accumulation of visceral fat leads to vascular damage and deposition of adipocytes into the fibroblasts of the transverse urethral muscle, resulting in chronic damage to the pelvic nerves, pelvic floor muscles, forced urethra, and sphincters significantly increasing the probability of stress incontinence in women.^[26]^ Increased visceral fat, which can lead to diabetes and other diseases, also plays a role in the development of female urinary incontinence.^[27]^ Ryan et al discovered that stress incontinence increased significantly with increasing body mass index and weight but decreased for all stress incontinence indicators with appropriate weight loss (5%–10%).^[28]^

Visceral fat is associated with an inflammatory response, which may affect the health and function of pelvic floor structures. According to that, Wang et al found that in female Zucker fatty rats, the thickness of the transverse muscle layer is noticeably diminished. Additionally, these rats exhibit a decrease in leak point pressure alongside an increased deposition of intracellular lipids within the urethral transverse muscle fibers.^[29]^

Our articles have these advantages. We chose a sufficiently large sample size, while the NHANES database was selected to be highly representative. In addition, we have added several confounding factors to make the data more reliable. Nevertheless, our research does have some constraints. The database data we analyzed were cross-sectional studies that could not determine causality. Therefore, we still need more sample data to prove causality. In addition, the data for our study was obtained from a U.S. public database, so it can only reflect the relationship between VAI and SUI among U.S. adult women.

## 5. Conclusion

We found a positive association between the VAI index and the likelihood of SUI prevalence. This study finds that controlling visceral fat growth may have a positive effect on the risk of SUI in women, but more studies are still needed to validate our findings.

## Author contributions

**Data curation:** Dakai Sun, Botao Xie, Yanqi Wang.

**Formal analysis:** Dakai Sun.

**Investigation:** Xingcheng Zhu, Haoyang He, Yanqi Wang.

**Resources:** Botao Xie.

**Writing – original draft:** Dakai Sun, Caie Hou, Xingcheng Zhu, Yanqi Wang.

**Writing – review & editing:** Dakai Sun, Caie Hou, Yanqi Wang.
